# Trimetallic Zeolitic Imidazolate Framework-Derived CoNiO_2_/NiCo_2_O_4_/NiFe_2_O_4_ Hierarchical Architecture: Unveiling Multi-Component Synergism for Ultrahigh-Capacity and Highly Stable Lithium Storage

**DOI:** 10.3390/molecules31050855

**Published:** 2026-03-04

**Authors:** Dingyuan Hu, Ningbo Yu, Wei Hua, Xuanyi Gao, Yuhong Luo, Yongbo Wu, Dong Shu, Lipeng Zhang

**Affiliations:** 1 School of Materials and New Energy, South China Normal University, Shanwei 516600, China; 2 School of Chemistry, South China Normal University, Guangzhou 510006, China; 3 Key Laboratory of Atomic and Subatomic Structure and Quantum Control (Ministry of Education), Guangdong Basic Research Center of Excellence for Structure and Fundamental Interactions of Matter, School of Physics, South China Normal University, Guangzhou 510006, China; 4 Guangdong Provincial Key Laboratory of Quantum Engineering and Quantum Materials, Guangdong-Hong Kong Joint Laboratory of Quantum Matter, South China Normal University, Guangzhou 510006, China

**Keywords:** polymetallic oxide, nano/micro hierarchical structure, metal–organic framework, lithium storage

## Abstract

Transition metal oxides (TMOs) have been recognized as highly prospective anode materials for lithium-ion batteries (LIBs) due to their low cost, high capacity, and distinctive lithiation mechanisms. Nevertheless, their practical adoption is constrained by significant volume changes during lithiation/delithiation, inferior electrical conductivity, severe particle agglomeration, unsatisfactory cycling stability, and limited rate performance. In an effort to mitigate these flaws, we developed a tactic employing a zeolitic imidazolate framework (ZIF) as the self-sacrificing template and tuning the Co/Fe/Ni ratio with a ZIF framework to prepare an innovative trimetallic metal–organic framework (MOF)-derived CoNiO_2_/NiCo_2_O_4_/NiFe_2_O_4_ compound (**CFNO422**) with nano/micro hierarchical architecture. The nano/micro hierarchical structure effectively accommodates volume changes, alleviates structural stress, and offers copious active sites for lithium storage. More importantly, the synergistic interaction among multiple component oxides promotes richer redox reactions and enhances electronic conductivity. Benefiting from the structural compatibility and composition, **CFNO422** delivers an outstanding reversible capacity (1301.3 mAh g^−1^ up to 120 cycles at 0.2 A g^−1^), enhanced rate capability (614.3 mAh g^−1^ even at 2.0 A g^−1^), and exceptional cycling stability (527.4 mAh g^−1^ over 600 cycles at 1.0 A g^−1^). This research proposes a versatile synthesis for MOF-derived polymetallic oxides as anode materials, opening a new avenue for advanced energy storage.

## 1. Introduction

Lithium-ion batteries (LIBs) have achieved predominance as an energy storage technology, thanks to their excellent cycle life, superior energy, and power density, and have found broad application in intelligent electronics, energy storage, and grid-scale energy storage [1]. However, commercial graphite anodes commonly employed in Li-ion battery exhibit a limited theoretical capacity of only 372 mAh g^−1^, which further degrades upon electrochemical cycling [2,3]. Graphite/silicon anodes have the potential to become a candidate for commercial graphite anodes. However, silicon/graphite anodes still have issues with unstable SEI in practical applications, and they can cause graphite to lose its inherent capacity after cycling. The short-term goal for their commercialization is to achieve a specific capacity of 500 mAh g^−1^, and the material price should meet the standard [4,5,6]. These limitations pose significant challenges in meeting the growing demands of the rapidly expanding LIB market. Considerable efforts have focused on developing novel superior energy density electrode materials [7]. Enhancing the energy density and cycling life of LIBs via such materials represents a viable approach to advancing high performance batteries and enabling their industrial scale applications [8,9]. Cost effective and readily available transition metal oxides (TMOs) such as NiO, Fe_2_O_3_, and Co_3_O_4_, and particularly ternary transition metal oxides (AB_2_O_4_, e.g., NiCo_2_O_4_, NiFe_2_O_4_, CoFe_2_O_4_), have already been recognized as prospective anode materials for LIBs [10,11,12,13,14,15]. Their appeal stems from a superior gravimetric specific capacity, which originates from distinctive lithiation mechanisms including conversion reactions such as Fe_2_O_3_, CuO, MnO, and/or alloying reactions (ZnO, GeO_2_, SnO_2_) [16]. However, TMOs and AB_2_O_4_ anodes inherently suffer from significant volume changes, poor electrical conductivity, severe particle agglomeration, and undesirable side reactions with the electrolyte during (de)lithiation [17,18]. These issues collectively result in inferior cycling stability, unsatisfactory rate performance, low coulombic efficiency, and rapid capacity decay, which have impeded their commercial adoption to date [19]. To address above-mentioned issues, extensive efforts have been conducted to overcome the inherent limitations. The first involves compositing electrode materials with electronically conductive buffer matrices, particularly carbon-based conductive components, ranging from carbon cloth and carbon nanotubes to reduced graphene oxide and carbon fibers, to enhance overall conductivity [20]. The second strategy entails synthesizing nanoscale structures to leverage their high specific surface area and favorable volume ratio, which facilitate rate performance by reducing ionic diffusion lengths [21]. The third strategy involves regulating material dimensions and morphology, such as constructing intricate hollow structures (eggshell, multi-shell and regulated interior functional structures), which could buffer volume changes during cycling and create rational contact points between the multifunctional active nanomaterial electrolyte [22,23]. This approach ensures conductivity integrity and ultra-long-term cycling stability. The fourth strategy involves designing multi-component active TMOs, where synergistic interactions between components improve reaction kinetics, enhance conductivity, and shorten charge transport channels. Moreover, multi-metallic units could yield richer redox reactions and superior electronic conductivity compared to bimetallic and monometallic ones [24].

Metal–organic frameworks (MOFs) are innovative porous nanomaterials with unique morphologies, assembled by combining organic components (organometallic complexes or organic ligands) with metal clusters or ions through covalent coordination bonds or other intermolecular interactions [25,26]. Due to their uniquely tunable topological structures, high specific surface areas as well as pronounced size-selectivity effects, MOFs have attracted extensive attention for diverse applications ranging from catalysis to energy storage, particularly in the latter [27,28]. MOFs are rarely used directly as battery materials due to inherent limitations including poor electronic conductivity, limited cycle life, significant irreversible capacity loss, and incomplete electrode reactions [29]. Comparatively, MOF composites and their derivatives retain the structural, porous, and compositional benefits of pristine MOFs while acquiring enhanced conductivity from incorporated functional components [30]. This synergistic improvement in electrochemical performance constitutes a promising strategy for the rational design of advanced battery materials. MOFs are regarded as promising sacrificial templates for synthesizing multi-component transition metal oxides (MTMOs) and multi-metallic oxides [29]. The phase homogeneity and chemical composition of MOFs could be precisely tailored through the meticulous regulation of calcination temperature, ambient atmosphere, the ratio of metal ions to ligands, metal species, and molar ratio [30]. Through the rational design of heterometallic MOFs incorporating two or more distinct metal ions, either homogeneous or hierarchically structured multi-component materials can be achieved. Importantly, MOFs can be transformed into polymetallic oxides with outstanding structural complexity and stability [31]. Furthermore, the tunable composition and architecture of MOFs enable their derivatives to afford abundant active sites, which enhance Li^+^ conversion kinetics and electrolyte infiltration performance, leading to superior electrochemical properties in LIBs [32]. For instance, Wang and collaborators synthesized porous Fe_2_O_3_@NiCo_2_O_4_ nanocages by calcining core-shell Ni-Fe-Co MOF precursors in air. When it reached to 100 cycles, this material exhibited the remarkable capacity of 1079.6 mAh g^−1^, which notably surpassed its theoretical capacity [33]. He et al. employed a stoichiometric regulation strategy to synthesize hierarchical ZnCo_2_O_4_ derived from Zn-Co Prussian blue analog (PBA). This material, characterized as micro-sized assemblies interconnected by nanoparticles, demonstrated superior performance (1353 mAh g^−1^ at 0.2 A g^−1^ after 200 cycles) in LIBs [34]. Xia and colleagues reported the synthesis of a trimetallic Fe-Co-Ni-MOF-74-derived Ni/CoFe_2_O_4_@C nanocomposite for the LIB anode possessing a distinctive porous architecture and a simple and direct one-pot pyrolysis method. The Ni/CoFe_2_O_4_@C could achieve a reversible capacity of 962 mAh g^−1^ at 0.1 A g^−1^ when it reached 50 cycles [35]. As anticipated, MOFs have been unequivocally established as optimal self-sacrificial templates to develop TMO anodes with significantly augmented cyclability. TMOs can be derived from MOF templates such as BDC/BTC-based MOFs, zeolite imidazole frameworks (ZIFs), Prussian blue and its analogues (PB and PBAs), etc. Particularly, ZIFs constitute a prominent subclass of MOFs, featuring a zeolite-like topology that is formed by the combination of nitrogen atoms and imidazole salts, which could encapsulate transition metal ions (Co^2+^, Zn^2+^, etc.) [36]. ZIFs combine the ultrahigh specific surface area and tunable porosity characteristic of MOFs with the inherent structural stability of zeolites, in addition to demonstrating other advantageous functional properties. In recent years, the well-defined architectures and facile synthesis of ZIFs have led to their growing utilization in rechargeable battery systems [37,38]. ZIFs and their derivatives have been successfully applied as anode materials in LIBs.

Moreover, the synthesis of multi-component TMOs presents substantial benefits. On the one hand, disparate redox potentials of the constituent metals can improve the efficiency of lithium storage and facilitate enhanced electron transfer kinetics, and synergistic interaction among multiple component oxides promotes richer redox reactions and enhances electronic conductivity [39,40]. On the other hand, the multi-component architecture within the TMOs is instrumental in mitigating the detrimental volume expansion/contraction in repeated charge/discharge cycling, significantly boosting the cycling durability of the battery [41]. Consequently, numerous researchers have focused on synthesizing multi-component nanostructured materials to harness the inherent synergistic effects for superior material performance. For instance, Niu and colleagues successfully synthesized the multi-component active metal oxide ZnO/ZnCo_2_O_4_/CuCo_2_O_4_ nanohybrid [42]. As an anode for LIBs, this nanohybrid demonstrated an outstanding capacity (1009 mAh g^−1^) at 3.0 A g^−1^ over 500 cycles while also exhibiting a remarkable rate capability, delivering 867 mAh g^−1^ even at 5.0 A g^−1^. Similarly, Xin and colleagues developed a novel yolk-shell structured Ni-NiCo_2_O_4_@ZnCo_2_O_4_ compound with a nanotetrahedron morphology using a facile co-precipitation technique [43]. Serving as the LIB anode, this material demonstrated outstanding reversible capacity (1097.5 mAh g^−1^) at 1.0 A g^−1^ over 600 loops as well as a superior rate capability, with 950.4 mAh g^−1^ maintained at 5.0 A g^−1^. These exceptional results were primarily assigned to the synergistic interaction among the constituent nanoparticles. In the synthesis of multi-component metal oxides, employing metal–organic frameworks (MOFs) as precursors or templates demonstrates unique advantages over the use of simple metal salts or conventional coordination compounds. Upon pyrolysis, MOFs directly transform into oxide composites characterized by high specific surface area and hierarchical porosity [37]. These structural features facilitate ion/electron transport and alleviate volume strain [44], while the porosity derived from MOFs offers greater diversity and compositional complexity compared to other porous materials [30]. Derivatives of multi-metallic MOFs provide enhanced tunability in controlling size, porosity, structure, and composition, thereby improving electronic/ionic conductivity and enriching redox chemistry at the desired potentials [32]. Studies indicate that MOF-derived multi-metallic oxides generally exhibit superior specific capacity and cycling stability in electrochemical energy storage compared to products from traditional methods due to close interfacial contact and synergistic effects among components, thus offering a robust platform for the rational design of high-performance electrode materials [24]. For example, Zhang et al. prepared various metal oxides using MOFs and synthesized corresponding transition metal oxides (TMOs) via simple coprecipitation without MOF derivation where the MOF-derived oxides demonstrated significantly better electrochemical performance [45]. Based on these compelling research findings, the utilization of MOFs as precursors for preparing multi-component materials presents a distinct and significant advantage for developing high performance LIB anodes.

Herein, we developed a facile MOF template strategy for the preparation of CoNiO_2_/NiCo_2_O_4_/NiFe_2_O_4_ with a unique nano/micro hierarchical structure, which is achieved through a combination of co-precipitation, stoichiometric tuning, and subsequent calcination under alternating air and nitrogen atmospheres. The nano/micro structure, composed of interconnected nanoparticles assembling into micro-sized assemblies, accommodates volume changes, alleviates structural stress, offers richer active sites in lithium storage, and ensures more efficient electron/ion transfer in the anode. More encouragingly, compared with bi-component oxides, synergistic interaction between multiple components oxides enables three active metal oxides to have lower electron transfer activation energy, generating more abundant redox reactions and superior electronic conductivity. Conferred by structural and compositional advantages, the as-prepared CoNiO_2_/NiCo_2_O_4_/NiFe_2_O_4_ sample demonstrated superior lithium storage performance, exhibiting outstanding cyclability and a remarkable rate performance. This research proposes a versatile synthesis approach for MOF-derived polymetallic oxides as outstanding electrode materials for energy storage and opens novel avenues for their future development and application.

## 2. Results and Discussion

### 2.1. Structure and Morphology Characterization

**Figure 1:**
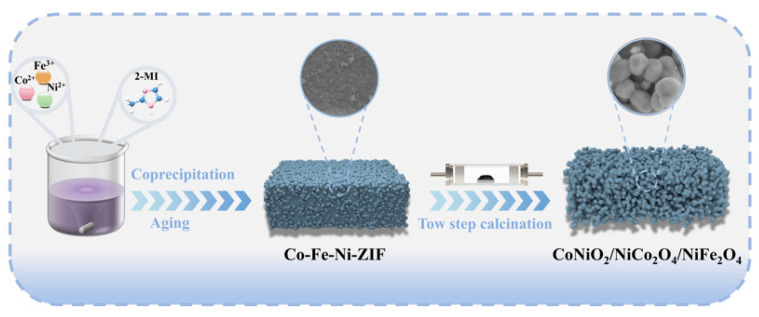
The schematic of the synthesis process for CoNiO_2_/NiCo_2_O_4_/NiFe_2_O_4_.

**Figure 2:**
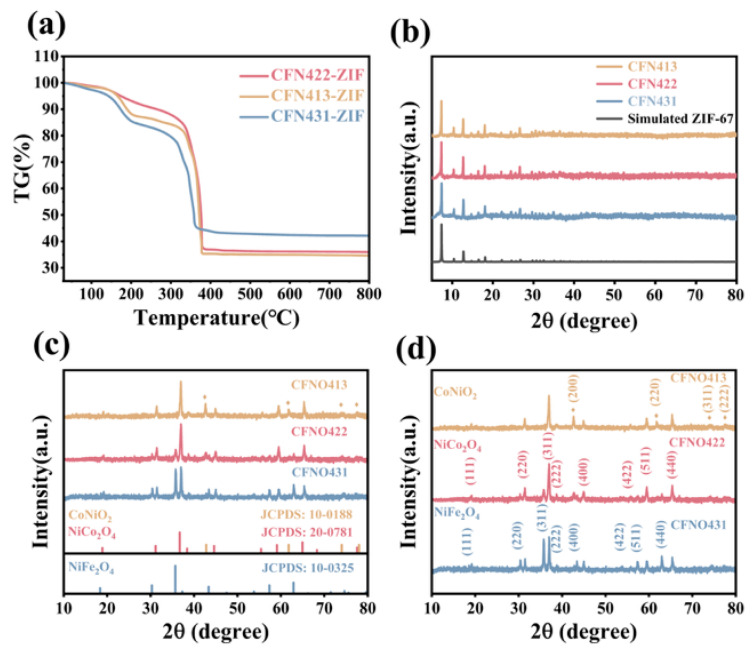
(**a**) TGA curve of the as-prepared **CFN422-ZIF**, **CFN413-ZIF**, and **CFN431-ZIF** under air atmosphere; XRD patterns of (**b**) **CFN413-ZIF**, **CFN422-ZIF**, **CFN431-ZIF**, and simulated **ZIF67**; and (**c**,**d**) XRD patterns of **CFNO422**, **CFNO413**, and **CFNO431**.

Three distinct oxide samples CoNiO_2_/NiCo_2_O_4_/NiFe_2_O_4_ (**CFNO422**), NiCo_2_O_4_/CoNiO_2_ (**CFNO413**), and NiCo_2_O_4_/NiFe_2_O_4_ (**CFNO431**) were synthesized respectively by adjusting the stoichiometric ratios of Co/Fe/Ni (4:2:2, 4:1:3, and 4:3:1) in the ZIF framework. Figure 1 illustrates the synthesis procedure of CoNiO_2_/NiCo_2_O_4_/NiFe_2_O_4_ derived from a trimetallic Co-Fe-Ni-ZIF (Co/Fe/Ni = 4:2:2). The synthesis of CoNiO_2_/NiCo_2_O_4_ and NiCo_2_O_4_/NiFe_2_O_4_ followed a similar protocol, with the only variation being the adjusted Co/Fe/Ni molar ratios within the ZIF precursor. Due to the robust coordination interaction of the ligand with the transition metal ions [46], a series of purple red trimetallic Co-Fe-Ni ZIFs precipitates were readily obtained (Figure S1) by mixing an aqueous solution of the 2-methylimidazole (2-MI) ligand, which contained the surfactant cetyltrimethylammonium bromide (CTAB), with an aqueous solution comprising varying proportions of Fe^3+^, Co^2+^, and Ni^2+^ ions at ambient temperature, followed by stirring and aging. The nitrogen-containing heterocyclic ligand 2-MI coordinates through its nitrogen atoms to metal ions, enabling concurrent enhancement of both porosity and functionality in the resulting Co-Fe-Ni-ZIF structure. We performed thermogravimetric analysis (TGA) on the three distinct Co/Fe/Ni-ZIF precursors in order to determine their optimal calcination temperature. Figure 2a presents the thermogravimetric analysis (TGA) curve of the **CFN422-ZIF**, **CFN413-ZIF**, and **CFN431-ZIF** precursors calcined in air, revealing two distinct weight-loss stages. The initial major weight loss observed at approximately 200 °C was primarily attributed to the removal of physisorbed water, crystalline water, and solvent evaporation. A significant mass loss in the range of around 400 °C was ascribed to the thermal decomposition of organic ligands and possibly the presence of surface-active agents. Subsequently, the weight remained nearly constant, indicating the formation of a stable crystalline phase. Based on this analysis, a two-step calcination strategy was employed for the **CFN422-ZIF**, **CFN413-ZIF**, and **CFN431-ZIF** precursors, involving initial calcination at 400 °C in air and subsequent treatment at 700 °C under a nitrogen atmosphere. Finally, a black product was obtained, in powder form (Figure S2). This dual-stage approach was designed to simultaneously maximize the specific surface area and enhance phase crystallinity in the MOF-derived TMOs composites [47].

The X-ray diffraction (XRD) patterns of the **CFN422-ZIF**, **CFN413-ZIF**, and **CFN431-ZIF** precursors are depicted in Figure 2b, which confirm their high crystallinity and zeolitic-type structure. XRD analysis for the **CFNO422**, **CFNO413**, and **CFNO431** samples confirmed their phase composition and crystal structure. As illustrated in Figure 2c,d, the diffraction peaks for **CFNO431** agreed with both spinel NiFe_2_O_4_ (JCPDS no. 10-0325) and NiCo_2_O_4_ (JCPDS no. 20-0781). For **CFNO413**, the diffraction peaks could be satisfactorily agreed with the cubic CoNiO_2_ (JCPDS no. 10-0188) and spinel NiCo_2_O_4_ (JCPDS no. 20-0781). When the Co/Fe/Ni ratio was adjusted to 4:2:2, the resulting sample **CFNO422** exhibited diffraction peaks of 18.4°, 30.3°, 37.3°, 43.4°, 47.5°, 53.8°, 57.4°, and 62.9°, which aligned with (111), (220), (311), (222), (400), (422), (511), and (440) planes of spinel NiFe_2_O_4_ (JCPDS no.10-0325), the diffraction peaks of 19.0°, 31.1°, 36.6°, 38.4°, 44.6°, 55.4°, 59.1°, and 64.9° corresponded to the (111), (220), (311), (222), (400), (422), (511) and (440) planes of spinel NiCo_2_O_4_ (JCPDS no. 20-0748), and the diffraction peaks of 42.8°, 61.8°, 74.0° and 78.0° corresponded to the (200), (220), and (311) planes of cubic phase CoNiO_2_ (JCPDS no. 10-0188). The phase composition of the synthesized materials were analyzed by refining the XRD patterns (Figure S3). Based on the refined results obtained from the XRD patterns, the primary crystalline phases in each sample could be identified. The analysis indicates that **CFNO422** has three coexisting crystal phases, while **CFNO413** and **CFNO431** have only two coexisting crystal phases.

**Figure 3:**
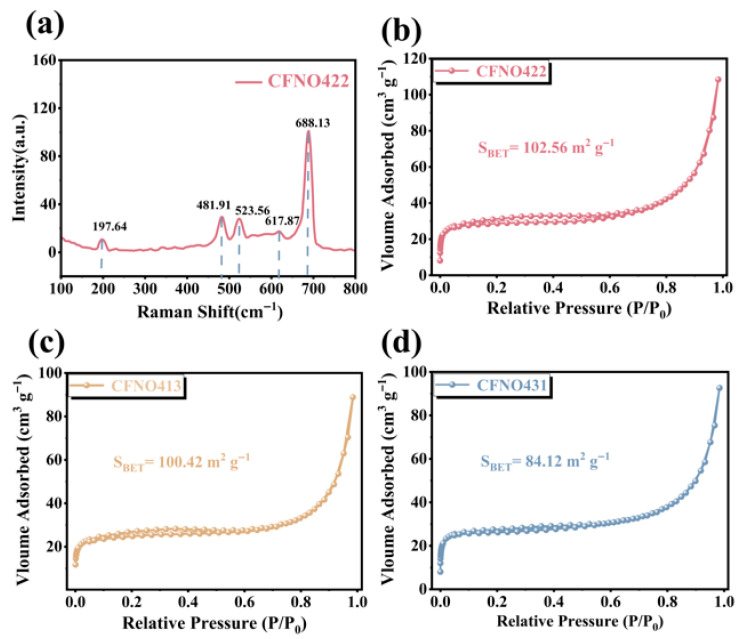
(**a**) The Raman spectrum of **CFNO422**. (**b**–**d**) N_2_ adsorption/desorption isotherms.

To further ascertain the precise composition of the **CFNO422** compound, Raman spectroscopy measurements were performed (Figure 3a). The Raman peaks observed at 197.64 and 481.91 cm^−1^ in the **CFNO422** material were assigned to the F_2g_ mode and the E_g_ mode of the spinel NiCo_2_O_4_, respectively. A broadened peak could be observed at 523.56 cm^−1^, which was indexed to the isostructural formation of CoNiO_2_ [48]. Furthermore, another prominent peak at 688.13 cm^−1^ was in agreement with the A_1g_ modes of NiFe_2_O_4_. A minor shoulder peak near 620 cm^−1^ was consistent with Co–O stretching of the A_1g_ mode for Co^2+^ at the tetrahedral (A) sites. This additional shoulder peak is highly indicative of a mixed spinel structure [49]. These results demonstrate the multi-component nature of **CFNO422** matching with the results of the XRD analysis.

To investigate the pore structure characteristics of **CFNO422**, **CFNO413**, and **CFNO431**, nitrogen adsorption–desorption measurements were conducted (Figure 3b–d). Based on Barrett–Joyner–Halenda (BJH) calculations, the Brunauer–Emmett–Teller (BET) surface areas of **CFNO422**, **CFNO413**, and **CFNO431** were recalculated as 102.56, 100.42 and 84.12 m^2^g^−1^, respectively. We also compared the specific surface areas of **CFNO422**, **CFNO413**, and **CFNO431** with other oxides (Table S1), and the synthesized **CFN422** exhibited a superior specific surface area. Correspondingly, ion-distribution pore size curves were also employed to compare the pore structures of these materials. As shown in Figure S4, the pore size distribution of all three samples centered around 6 nm, with **CFNO422** exhibiting superporosity. Porous structures promote abundant electrochemically active sites, buffer volume expansion, and enhance ion/charge diffusivity. Consequently, large surface areas and pronounced porosity can shorten the diffusion pathways for electrolyte/lithium transfer, thereby improving electrochemical kinetics and rate capability, which can be further validated by electrochemical measurements.

**Figure 4:**
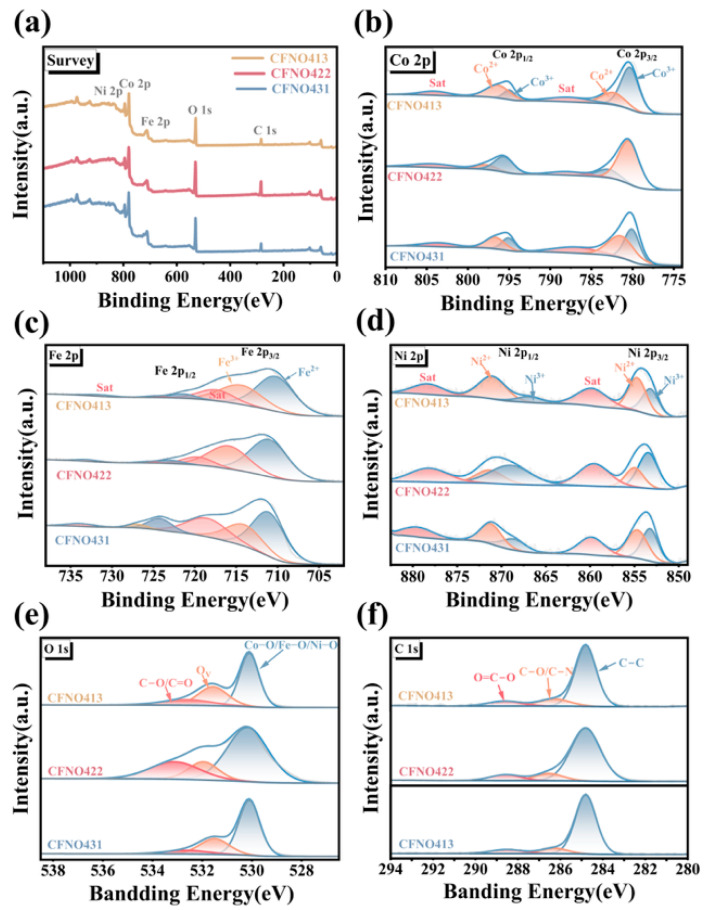
(**a**) XPS survey; (**b**) Co 2p; (**c**) Fe 2p; (**d**) Ni 2p; (**e**) C 1s; (**f**) O 1s.

Elemental composition and surface chemical states of the as-prepared samples were analyzed by X-ray photoelectron spectroscopy (XPS), as depicted in Figure 4a–f. The resultant survey spectra of the as-prepared products conclusively confirmed the existence of C, O, Fe, Ni, and Co elements. The high-resolution Co 2p spectra were deconvoluted into six characteristic subpeaks (Figure 4b). Peaks observed in 780.44 eV and 795.59 eV aligned with the Co^3+^ state, belonging to the Co 2p_3/2_ and Co 2p_1/2_ doublet; separately, those appearing at 782.94 eV and 797.77 eV could agree with Co^2+^, which belong to the Co 2p_3/2_ and Co 2p_1/2_ doublet. Furthermore, two satellite peaks, indicative of the Co^2+^ oxidation state, were discerned at 787.92 eV and 804.17 eV [50,51]. The high-resolution Fe 2p spectra (Figure 4c) also fitted into six component peaks whose peaks were situated at approximately 722.85 eV and 711.07 eV, which agree with an Fe 2p_1/2_ and Fe 2p_3/2_ doublet existing with a separation (ΔE_Fe_) of 11.78 eV, confirming the existence of the Fe^2+^ state. Peaks observed at 715.89 eV and 725.85 eV aligned with Fe^3+^, signifying that a higher Fe^3+^ oxidation state existed in the composite structure [52,53]. The high-resolution Ni 2p photoelectron spectra are depicted in Figure 4d. Peaks at 853.66 eV and 855.27 eV unequivocally revealed the existence of Ni^2+^ and Ni^3+^ in the Ni 2p_3/2_ region. A satellite peak for Ni 2p_3/2_ at 860.02 eV was exhibited. The Ni 2p_1/2_ region showed peaks at 869.89 eV and 872.36 eV, aligning with Ni^2+^ and Ni^3+^, respectively, and the corresponding satellite peak appeared at 879.49 eV [54]. Peak-fitting of the O 1s spectrum yielded three component peaks at 533.10 eV, 531.95 eV, and 530.17 eV (Figure 4e), corresponding to surface adsorbed species (C=O/C–O), lattice defects (specifically, oxygen vacancies), and the metal–oxygen bonds (Co–O/Fe–O/Ni–O) within the crystal lattice, respectively [55]. This analysis substantiates the presence of oxygen vacancies. Furthermore, three component peaks were observed in the high-resolution C 1s spectrum (Figure 4f), assigned to O–C=O functional groups, C–N bonds, and graphitic carbon [51,55].

**Figure 5:**
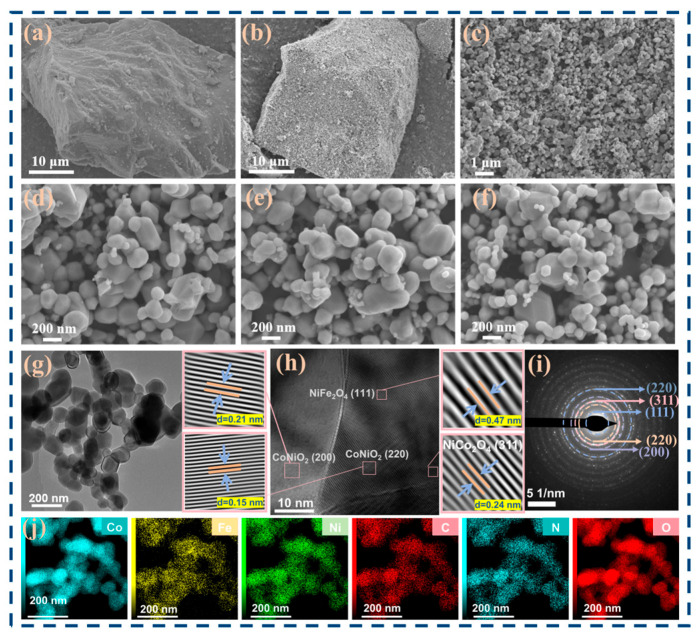
SEM pictures of (**a**) the precursor for **CFN422-ZIF**, (**b**–**d**) **CFNO422**, (**e**) **CFNO413**, and (**f**) **CFNO431**; (**g**) TEM image of **CFNO422**; (**h**) HRTEM image of **CFNO422**; (**i**) SAED pattern and (**j**) elemental mapping images for **CFNO422**.

The characterization of external structure and morphology for the as-prepared samples (**CFNO422**, **CFNO413**, and **CFNO431**) were performed using scanning electron microscopy (SEM, ZEISS Ultra 55, Carl Zeiss AG, Oberkochen, Germany). We clearly observed that all samples manifested as agglomerates of nanoparticles, which is also relatively common in transition metal oxides [56,57]. As presented in Figure 5a and Figure S5a, the **CFN422-ZIF** precursor exhibited a micro-scale assembled architecture assembled from interconnected, cobblestone-like nanoparticles. Then, the calcination procedure facilitated the recrystallization of the **CFN422-ZIF** precursor, and resulting **CFNO422** sample displayed a nano/micro hierarchical architecture (Figure 5b–d) that preserved the morphology of the **CFN422-ZIF** precursor. Specifically, **CFNO422** exhibited plate-like micro-assemblies consisting of numerous smooth and interconnected nanoparticles with diameters ranging from approximately 100 to 400 nm. Moreover, there were large gaps between the particles. The architecture of **CFNO422** is highly advantageous for facilitating electrolyte infiltration and enhancing lithium-ion diffusion kinetics. In contrast, Figure 5e and Figure S5b show that the **CFNO413** sample was characterized by cobblestone-like nanoparticles with a larger average particle size and a comparatively rougher surface texture, which impedes electrolyte infiltration and elongates the lithium-ion transport pathways, consequently resulting in sluggish ionic transport kinetics. Simultaneously, **CFNO431** presented as monodisperse pebble-like particles that had smooth surface without no obvious collapse, while those particles exhibited irregular sizes (Figure 5f and Figure S5c), which hinders ion transport. Concurrently, the internal microstructure of **CFNO422** was examined using transmission electron microscopy (TEM, FEITalos F200x, Thermo Fisher Scientific, Hillsboro, OR, USA). TEM imaging of **CFNO422** further clearly revealed an interconnected nanoparticle configuration, with substantial voids between nanoparticles (Figure 5g). The HRTEM image of **CFNO422** (Figure 5h) showed distinct lattice fringes with measured spacings of 0.21 nm and 0.15 nm, matching the (200) and (220) planes of CoNiO_2_ [58,59], respectively, while lattice fringes with spacings of 0.24 nm and 0.48 nm could be assigned to the (311) plane of NiCo_2_O_4_ and the (111) plane of NiFe_2_O_4_ [60,61], respectively, revealing the ternary mixed metal oxide was successfully synthesized and agreed with the results of the XRD analysis. The SAED pattern (Figure 5i) further corroborated the crystalline structure of **CFNO422**, with diffraction rings agreeing with the (220) and (200) planes of CoNiO_2_, diffraction rings agreeing with the (111) and (311) planes of NiFe_2_O_4_, and diffraction rings agreeing with the (220) plane of NiCo_2_O_4_. Finally, elemental mappings of **CFNO422** (Figure 5j) revealed that Co, Fe, Ni, O, N, and C were uniformly distributed. It is noteworthy that the elemental ratios measured by EDS exhibited significant deviations from the initial feed ratios (Figure S6). This indicates that during ZIF formation and subsequent calcination, different metal ions demonstrate distinct incorporation behaviors and varying degrees of coordination affinity with dimethylimidazole.

### 2.2. Electrochemical Properties

**Figure 6:**
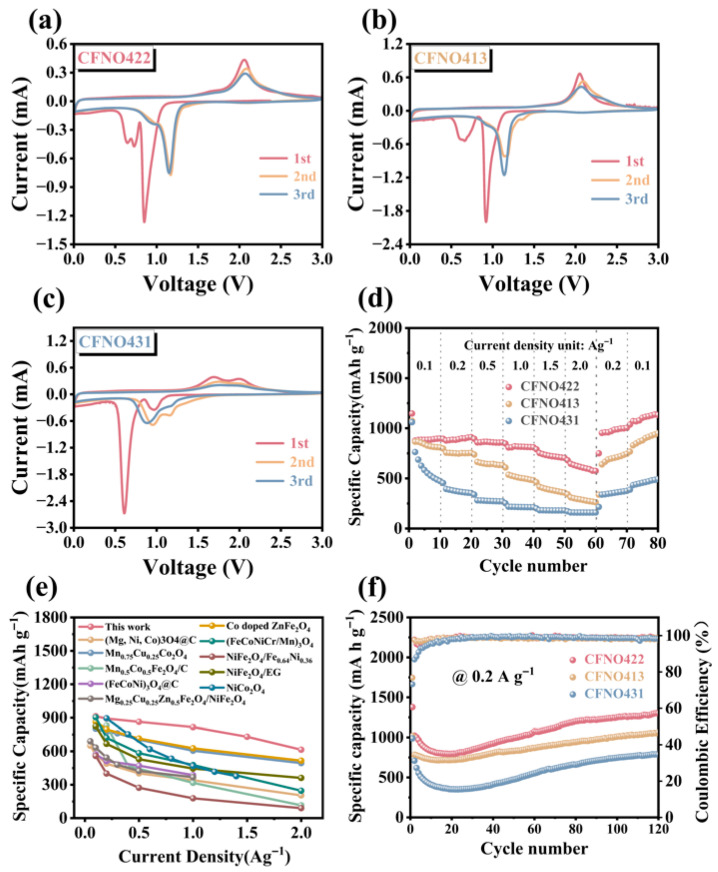
CV profiles of (**a**) **CFNO422**, (**b**) **CFNO413**, and (**c**) **CFNO431** at the sweeping rate of 0.1 mV s^−1^; (**d**) rate performance of **CFNO422**, **CFNO413**, and **CFNO431**; (**e**) the comparison of rate performances for **CFNO422** anode and oxide materials reported before; (**f**) cycling performances of three **CFNO422** electrodes at 0.2 A g^−1^.

For evaluating the electrochemical performance and lithium storage capacity of the CoNiO_2_/NiCo_2_O_4_/NiFe_2_O_4_ (**CFNO422**) composite material and the reference samples CFNO413 and CFNO431 as LIB anode materials, cyclic voltammetry (CV) measurements were conducted on **CFNO422**, **CFNO413**, and **CFNO431** with a sweeping rate of 0.1 mV s^−1^ (Figure 6a). For the first cathodic sweeping, CFNO422 exhibited distinct reduction peaks appearing at 0.65 V, 0.73 V, and 0.85 V, which were ascribed to the reduction of NiCo_2_O_4_, NiFe_2_O_4_, and CoNiO_2_ into corresponding metallic states (Co, Fe, and Ni), lithium intercalation as well as the formation of Li_2_O and the solid electrolyte interphase (SEI) [57,62]. Two oxidation peaks emerged at 1.5 V–2.1 V during the anodic scan, indicating the oxidation of Fe to Fe^3+^, Co to Co^3+^, and Ni to Ni^2+^ [63]. These features confirm that **CFNO422** enables reversible lithium insertion/extraction via an oxidation–reduction mechanism. For the CV curves of **CFNO413** and **CFNO431** (Figure 6b,c), two reduction peaks were displayed at approximately 0.66 V and 0.96 V during the first cycle, and two oxidation peaks between 1.5 V and 2.1 V. Notably, the CV curves obtained for **CFNO422** showed good overlap between the second and third measurements, indicating highly reversible electrochemical reactions compared with the counterparts, which reflected superior electrochemical reversibility and stability during lithiation/delithiation [64]. Additionally, for three electrodes, a reduction peak was observed at approximately 0.01 V, which could be explained by the reduction of metal oxides and the subsequent formation of zero-valent metal nanoparticles embedded in the Li_2_O matrix [65]. Moreover, the first cycle exhibited distinct differences from the subsequent cycles, indicating the occurrence of irreversible reactions [35]. Based on the CV results and in conjunction with the prior reports, we speculate that the electrochemical behavior for the as-synthesized materials can be described by the reaction equations [53,66]:

NiCo_2_O_4_ + 8Li^+^ + 8e^−^ → Ni + 2Co + 4Li_2_O (1)

NiFe_2_O_4_ + 8Li^+^ + 8e^−^ → Ni + 2Fe + 4Li_2_O (2)

CoNiO_2_ + 4Li^+^ + 4e^−^ → Co + Ni + 2Li_2_O (3)

Ni + Li_2_O ↔ NiO + 2Li^+^ + 2e^−^ (4)

2Fe + 3Li_2_O ↔ Fe_2_O_3_ + 6Li^+^ + 6e^−^ (5)

2Co + 2Li_2_O ↔ 2CoO + 4Li^+^ + 4e^−^ (6)

2CoO + 2/3Li_2_O ↔ 2/3 Co_3_O_4_ + 4/3 Li^+^ + 4/3 e^−^ (7)

Figure S7a–c shows the charge/discharge profiles of the **CFNO422**, **CFNO413**, and **CFNO431** electrodes for the initial three cycles at 0.2 A g^−1^. Distinct plateaus at 0.52–0.75 V and 0.75–1.25 V were observable on the discharge profiles and a broad sloping plateau at 1.5–2.5 V on the charge profiles, which aligned with the CV profiles. The initial large discharge/charge capacities of **CFNO422** (1377.9/1058.8 mAh g^−1^) contributed to the formation/dissociation of gel-like polymeric films in the active material surface as well as incomplete delithiation resulting from electrode structure rearrangement [67,68]. The initial coulombic efficiency was 76.8%. Furthermore, the capacity loss during the initial cycle was mainly ascribed to the generation of irreversible SEI films and Li_2_O [69]. For the second and third cycles, the coulombic efficiencies of **CFNO422** were 97.8% and 95.2%. Meanwhile, **CFNO13** and **CFNO431** exhibited initial discharge/charge capacities of 1018.3/781.5 mAh g^−1^ and 988.52/723.5 mAh g^−1^, respectively, with corresponding coulombic efficiencies of 76.74% and 73.2%, respectively. Obviously, among the three as-synthesized electrodes, **CFNO422** exhibited a higher lithium storage capacity. Interestingly, during the initial cycling phase, discharge capacity exhibited by the discharge branch was observed to exceed that of the corresponding charge branch. This indicates the presence of additional, partially reversible processes during lithiation. The nano-micro hierarchical structures and the existence of multiphase oxides, coupled with electrolyte decomposition and the formation of the solid electrolyte interphase (SEI), could provide additional discharge capacity [45,51].

**Figure 7:**
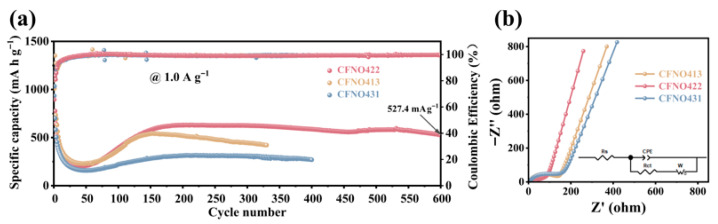
(**a**) Long-term cycling stability of **CFNO422** at 1.0 A g^−1^; (**b**) tests of the electrochemical impedance spectra (EIS) for the as-prepared **CFNO422**, **CFNO413**, and **CFNO431** electrodes were performed at frequencies from 0.01 Hz to 100,000 Hz.

The rate performance can be used to clarify the durability of fast charge and discharge cycles. Figure 6d depicts the rate performances of **CFNO422**, **CFNO413**, and **CFNO431** anodes ranging from 0.1 to 2 A g^−1^. Evidently, **CFNO422** demonstrated the most favorable rate performance; reversible capacities of 913.2, 892.8, 863.4, 816.5 and 730.3 mA h g^−1^ were attained at 0.1, 0.2, 0.5, 1.0, and 1.5 A g^−1^, respectively, even at 2.0 A g^−1^, a capacity of 614.3 mAh g^−1^ could be sustained. Remarkably, the capacity of **CFNO422** recovered to 1105.8 mAh g^−1^, then went back to 0.1 A g^−1^, and further increased with subsequent cycling. This underscores the exceptional rate performance of the **Li/CFNO422** battery, markedly surpassing the performance of electrodes based on **CFNO413** and **CFNO431**, which was primarily ascribed to the nano/micro hierarchical architecture, which can offer cushion space to tolerate structural strain and shortens the ion transport path and the synergistic effect between the multiple components that provide electrical conductivity and wealthier redox chemical kinetics [55,69]. Furthermore, the rate performance of **CFNO422** in LIBs was demonstrably superior to that of other reported comparable materials (Figure 6e). The long-term cycling stability of the **CFNO422**, **CFNO413**, and **CFN431** electrodes is illustrated in Figure 6f. At a low current density of 0.2 A g^−1^, **CFNO422** exhibited an exceptional capacity (1301.3 mAh g^−1^) compared to **CFNO413** (1064.2 mAh g^−1^) and **CFNO431** (787.4 mAh g^−1^) over 120 cycles. More strikingly, at 1 A g^−1^, the **CFNO422** anode maintained a superior capacity (527.4 mAh g^−1^) over 600 cycles (Figure 7a). Even the coulombic efficiency of **CFNO422** could be kept at roughly 100% during the 600 cycling procedures, verifying an outstanding long cycling performance. The outstanding cycling performance of **CFNO422** was also better than that of the reported metal oxide anodes for LIBs (Table S2). A capacity decay observed during long-term cycling at high current densities was possibly due to some irreversible side reactions such as electrolyte decomposition and depletion as well as unavoidable volume expansion during lithiation/delithiation processes [65]. Interestingly, the capacity of **CFNO422** showed an increasing trend upon cycling at both high and low current densities, a phenomenon commonly observed in transition metal oxide anode materials. This reversible capacity growth may be attributed to: (i) during the lithiation process, the transition metal species could be progressively reactivated, which could expose more Co, Fe, and Ni active sites [70]; (ii) interfacial storage facilitated by a dual-phase capacitive mechanism at the Co/Li_2_O, Fe/Li_2_O, and Ni/Li_2_O interfaces, where Li^+^/electron accumulation occurs at the Li_2_O/transition metal side, thereby promoting interfacial charge transfer [71]; (iii) activating electrolyte degradation can lead to a reversible decomposition and formation of polymer gel-like films, which could provide extra pseudocapacitive contributions [55,72]; (iv) the unique hierarchical architecture and well-dispersed nanostructure offer continuous electron pathways, significantly shortening the charge diffusion length, which facilitates electron transport in **CFNO422** and mitigates volume variations during cycling [55]; (v) oxide anode materials undergo phase transitions or structural rearrangements during cycling, forming new phases or defect structures. These new structures may exhibit higher lithium-ion storage capacity [45]; (vi) the solid electrolyte interphase (SEI) film is a protective layer formed on the surface of the anode in lithium-ion batteries. Its composition and properties may change during the cycling process. The decomposition or reconstruction of the SEI film may release additional lithium ions or provide more efficient pathways for lithium ion transport [71], thereby increasing the battery’s capacity; and (vii) it may be related to the growth of polymer or gel-like thin films around metal nanoparticles at low discharge voltage as well as the surface capacitance of transition metal particles based on the space charge storage mechanism [67]. The EIS measurements were performed on the as-prepared anode materials (Figure 7b). In a low frequency region, the Nyquist plots of the **CFNO422**, **CFNO413**, and **CFNO431** electrodes exhibited a sloping line associated with Warburg impedance (W_s_) regarding lithium-ion diffusion; a semicircle also appeared in the mid-frequency zone linked with the charge transfer resistance (R_ct_) [69,73]. Notably, **CFNO422** displayed a smaller semicircle diameter and a steeper line slope, indicating enhanced charge diffusion kinetics.

**Figure 8:**
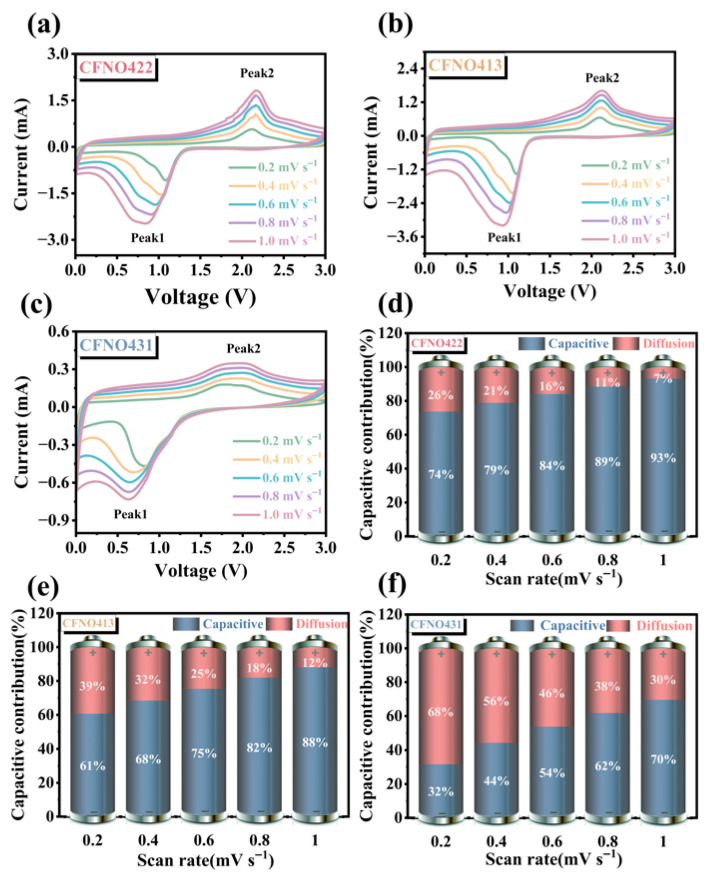
The CV curves at various scan velocities for (**a**) **CFNO422**, (**b**) **CFNO413**, and (**c**) **CFNO431**; The pseudocapacitance contribution at various scanning rates for (**d**) **CFNO422**, (**e**) **CFNO413**, and (**f**) **CFNO431**.

Cyclic voltammetry (CV) was carried out at sweep velocities of 0.2, 0.4, 0.6, 0.8, and 1.0 mV s^−1^ (Figure 8a–c) to investigate the electrochemical reaction kinetics of the as-synthesized materials. All redox peaks showed persistent fluctuations, which revealed that the redox peaks constantly rose with the incremental scan rate. To quantitatively elucidate the connection between the current and the sweeping rate and determine the contribution of pseudocapacitive behavior, the following equation was employed [54]:

(8) $$i=a\;v^{b}$$

(9) logi=blogv+loga

(10) $$i=k_{1}v+k_{2}v^{1/2}$$

The voltametric response for the electroactive material could be assessed via the logarithmic connection amid the peak current (i) and the scan rate (v): logi=b logv+loga. Calculated slope (b) values delineated two critical limiting cases: *b* = 0.5 corresponds to a Faradaic insertion process governed by semi-infinite linear diffusion, and b = 1.0 indicates a capacitive process where charge storage is independent of ion diffusion kinetics [74]. As illustrated in Figure S8, For the **CFNO422** electrode, the resulting calculated b values were 0.51 and 0.73, respectively, notably exceeding those of the two other reference samples, indicating a synergistic interplay between the electrode materials’ inherent capacitive behavior and its ion migration kinetics, which is pivotal for augmenting the overall charge storage capability of the anode. In Equation (10), $k_{1}v$ corresponds to the pseudocapacitive capacity, while $k_{2}v^{1/2}$ represents the diffusion-controlled capacity. The detailed fitting and quantification of the capacitive contribution ratio across various scan rates (Figure S9a–c) revealed that the pseudocapacitive contribution of **CFNO422** substantially increased up to 93.16% compared to 88.14% and 69.57% for **CFNO413** and **CFNO431** at 1 mV s^−1^, respectively. This significant enhancement is likely attributable to the optimized composition and structural regulation in **CFNO422** encompassing synergistic interactions among the multiple components, the unique micro/nano hierarchical architecture, the presence of oxygen vacancies, shortened diffusion pathways, low mass-transfer resistance, and abundant interparticle voids. Interestingly, the pseudocapacitive contribution of all electrodes increased with the increase in sweeping rates, with the **CFNO422** electrode being the most pronounced.

**Figure 9:**
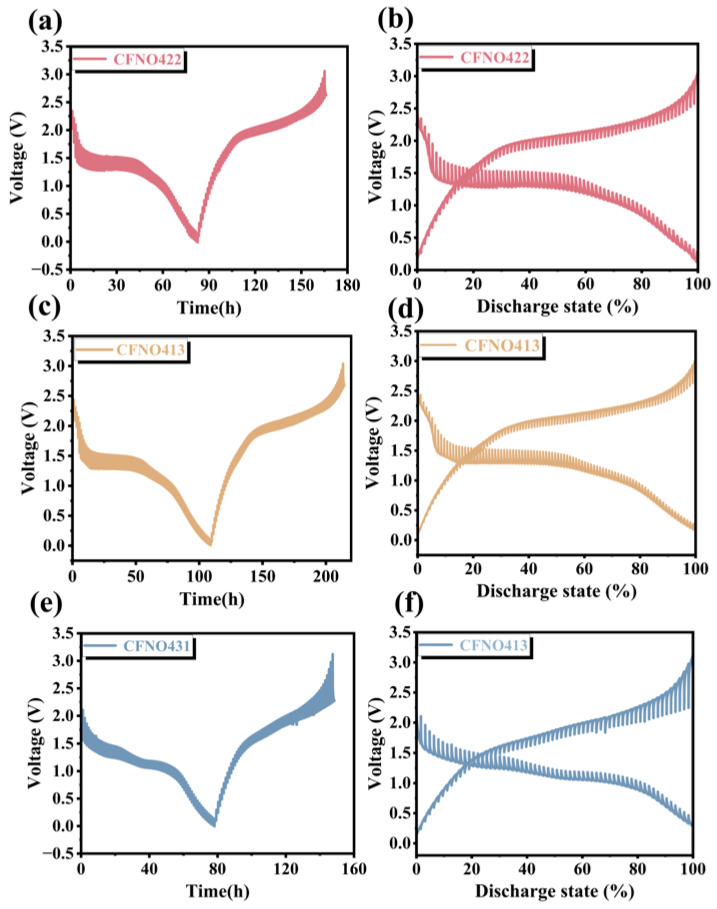
GITT titration curve for (**a**) **CFNO422**. Discharge/charge profile for (**b**) **CFNO422**. GITT titration curve for (**c**) **CFNO413**. Discharge/charge profile for (**d**) **CFNO413**. GITT titration curve for (**e**) **CFNO431**. Discharge/charge profile for (**f**) **CFNO431**.

Moreover, GITT analysis substantiated the superior Li-ion diffusion coefficient of **CFNO422** compared to **CFNO413** and **CFNO431** (Figure 9a–f and Figure S10). This analysis records the voltage responses during the second cycle of the titration process. A discernible voltage shift during each relaxation step reflects the overpotential value indicating the extent of polarization [41]. Furthermore, a lower overpotential thus corresponds to reduced polarization and enhanced lithium-ion diffusion kinetics. The chemical diffusion coefficient of Li^+^ (D_Li+_) was directly evaluated from the equation [45]:

(11) $$D_{{\mathrm{Li}}^{+}}=\frac{4}{\pi \tau }{\left(\frac{{m_{B}V}_{M}}{M_{B}A}\right)}^{2}{\left(\frac{{∆E}_{S}}{{∆E}_{\tau }}\right)}^{2}$$

**Figure 10:**
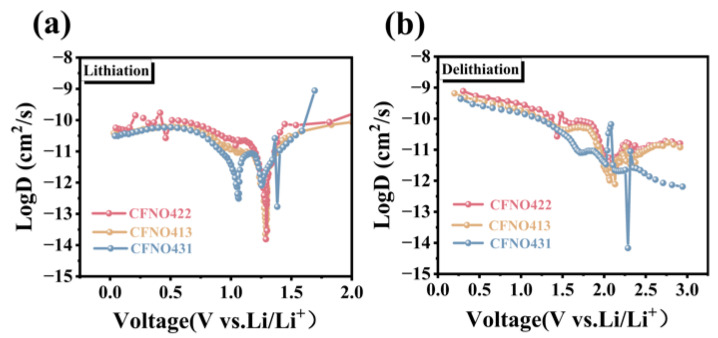
Calculated Li^+^ diffusion coefficients upon the (**a**) lithiation and (**b**) delithiation process of **CFNO422**, **CFNO413**, and **CFNO431** via the GITT test.

In this equation, τ is defined as the duration of the constant current pulse. m_B_, V_M_, M_B_, and A correspond to the mass, molecular volume, molecular mass, and electrode surface area of the electroactive species, respectively. Furthermore, $∆E_{S}$ and $∆E_{\tau }$ signify the voltage change observed during the pulse process and the relaxation voltage variation associated with the corresponding lithiation/delithiation steps. The GITT titration curves for **CFNO422**, **CFNO413**, and **CFNO431** are depicted in Figure 9a,c,e, whereas the corresponding GITT profiles for the voltage expression are presented in Figure 9b,d,f. A similarity was observed among the three curves, suggesting an identical reaction mechanism across all samples. As depicted in Figure 10a,b, **CFNO422** exhibited the significantly highest lithium-ion diffusion coefficient (D_Li+_) and demonstrated outstanding electrochemical kinetics compared with the **CFNO413** and **CFNO431** electrodes, which corresponded to the EIS results. Intriguingly, the D_Li+_ values for all samples first exhibited a decline, then an increase, and finally declined again. The phenomenon above-mentioned occurs due to an “activation process” where the electrodes are activated, presenting an increase in D_Li+_ values. During the lithiation process, the active material undergoes a phase transformation from a single phase to a multiphase state. This phase change induces a significant evolution in the crystal structure. The resultant structural rearrangement impedes Li^+^ migration, leading to a reduction in the D_Li+_ values [51,65].

## 3. Experimental

### 3.1. Chemicals

The primary chemical reagents employed were cobalt(II) nitrate hexahydrate (Co(NO_3_)_2_·6H_2_O, 99.0%, Macklin, Shanghai, China), iron(III) chloride hexahydrate (FeCl_3_·6H_2_O, 99%, Sigma-Aldrich Chemical Co, Shanghai, China), nickel(II) nitrate hexahydrate (Ni (NO_3_)_2_·6H_2_O, 99.0%, Macklin, Shanghai, China), 2-methylimidazole (99.0%, Macklin, Shanghai, China), and cetyltrimethylammonium bromide (CTAB, 98%, Macklin, Shanghai, China). All reagents were utilized as acquired with no additional purification.

### 3.2. Material Synthesis

Synthesis of Co-Fe-Ni-ZIF precursors: In a facile synthesis protocol, the Co-Fe-Ni-ZIF precursor was directly synthesized via a liquid-phase diffusion method. Firstly, 1 mmol of Co(NO_3_)_2_·6H_2_O, 0.5 mmol of FeCl_3_·6H_2_O, and 0.5 mmol of Ni(NO_3_)_2_·6H_2_O were completely dissolved in deionized water (DI water, 20 mL) containing a requisite amount of cetyltrimethylammonium bromide (CTAB). The mixture pared above was subsequently agitated at room temperature until a clear purple black solution was obtained. In another beaker, 4.5 g of 2-methylimidazole (2-MI) was dissolved in DI water (70 mL). Then, the purple black metal salt solution was swiftly introduced into the clarified solution of 2-methylimidazole under continuous stirring. After agitating for approximately 1 h, this mixture solution was subsequently allowed to age for several hours. The collected purple precipitate was successfully obtained with centrifugal force, then the sediment was cleaned multiple times with ethanol. This was finally dried overnight in an oven setting temperature to 60 °C to yield the precursors Co-Fe-Ni-ZIF (labeled as **CFN422-ZIF**). Simultaneously, using the same procedure, two additional precursors were prepared by tuning the molar ratios of Co, Fe, and Ni to 4:1:3 and 4:3:1, denoted as **CFN413-ZIF** and **CFN431-ZIF**, respectively.

Synthesis of CFNO: The **CFN422-ZIF**, **CFN413-ZIF** and **CFN431-ZIF** precursors were positioned in a tube furnace with two-stage thermal treatment. Initially, the obtained products were calcined at 400 °C with a controlled heating rate of 3 °C/min in an air atmosphere. Following this initial oxidative step, the materials underwent a second annealing at 700 °C in a N_2_ atmosphere, yielding the final products designated as **CFNO422**, **CFNO413**, and **CFNO431**, respectively.

### 3.3. Material Characterization

X-ray diffraction (Bruker D8 diffractometer, Bruker Company, Billerica, MA, USA) utilizing a Cu Kα radiation source (λ = 1.5418 Å) was used to collect the XRD patterns. Thermogravimetric analysis (TGA) was executed using a NETZSCH TG 209 F1 instrument (NETZSCH-Gerätebau GmbH, Selb, Germany), and the measurement was conducted in an air atmosphere from 30 to 800 °C with controlled heating rate of 10 °C/min. The LabRam HR Evolution spectrometer (Horiba Scientific, Palaiseau, France) was employed to acquire the Raman spectra. X-ray photoelectron spectroscopy (XPS, Al Kα) was used to analyze the surface elemental composition and chemical states of the materials. The structure and morphology of the sample were performed through scanning electron microscopy (SEM, ZEISS Ultra 55, Carl Zeiss AG, Oberkochen, Germany) and high-resolution transmission electron microscopy (HR-TEM, FEITalos F200x, Thermo Fisher Scientific, Hillsboro, OR, USA). The high-angle annular dark-field (HAADF)-STEM was used to reveal detailed structural information.

### 3.4. Electrochemical Testing

CR2032 coin-type cells were assembled in a glovebox filled with argon with low contents of oxygen and water maintained. The anode electrodes could be obtained by mixing 80 wt% of the as-synthesized **CFNO422**/**CFNO413**/**CFNO431**, 10 wt% of poly-vinylidene fluoride, and 10 wt% of acetylene black. The mixture was distributed in N-methyl-2-pyrrolidone followed by continuous agitation overnight at 25 °C. The homogeneous slurry was uniformly coated on copper foil utilizing a 100 μm scraper. The coated electrodes were placed in vacuum drying oven setting at 110 °C for 12 h and then cut into circular pieces (12 mm in diameter); the loading amount of active substance is approximately 0.88 mg cm^−2^. The separator was a polypropylene (PP) membrane (19 mm in diameter). The electrolyte was formulated in a 1:1:1 (by volume) mixed solvent of ethylene carbonate (EC), diethyl carbonate (DEC), and ethyl methyl carbonate (EMC) with 1.0 M LiPF_6_. The lithium metal served as the counter electrode. Subsequently, the LANDdt system (CT 2001A, Wuhan LAND Electronic Co. Ltd., Wuhan, China) was used to conduct galvanostatic charge/discharge (GCD) tests, galvanostatic intermittent titration technique (GITT) measurements, long cycling tests, and rate performance tests (0.01–3.0 V). An electrochemical workstation (CHI-760E, CH Instruments Inc., Shanghai, China) was used to analyze the electrochemical properties via the electro-chemical impedance spectroscopy (EIS) test, cyclic voltammetry (CV) curves, and pseudocapacitance test.

## 4. Conclusions

In summary, we devised a facile synthetic strategy involving the stoichiometric regulation of the Co:Fe:Ni ratio within the zeolitic imidazolate framework (ZIF) to successfully synthesize NiCo_2_O_4_/NiFe_2_O_4_/CoNiO_2_ (**CFNO422**). This material possesses a unique nano/micro hierarchical architecture, manifesting as a nano/micro hierarchical particulate architecture where interconnected nanoparticles are assembled into micro-scale architectures. Due to its distinct compositional and structural advantages, **CFNO422** exhibits superior electrochemical performance. Specifically, the **CFNO422** anode maintained a capacity of 1301.3 mAh g^−1^ up to 120 cycles at 0.2 A g^−1^, and exhibited excellent cycling stability (527.4 mAh g^−1^ at 1.0 A g^−1^ over 600 cycles). Furthermore, it presented an exceptional rate performance (614.3 mAh g^−1^ under high-rate cycling at 2 A g^−1^). Systematic investigation into the lithium storage kinetics revealed that the polymetallic synergistic effect effectively buffers the significant volume changes encountered during cycling and provides robust cohesion within the multi-oxide structure. More encouragingly, compared with bi-component oxides, the synergistic effects of multiple component oxides enable the three active metal oxides to have lower electron transfer activation energy, generating more abundant redox reactions and superior electronic conductivity. They all further improved the superior rate performance and lithium storage properties. Moreover, the constructed nano/micro hierarchical architecture significantly accommodates volume changes, alleviates structural stress, enhances charge transfer, accelerates lithium-ion diffusion, boosts interfacial activity, offers more richer active points for lithium storage, and ensures the quick transformation of electrons/ions in the electrode. These characteristics can achieve both structural stability and cycling stability. This research provides significant insight into the controlled synthesis of trimetallic oxides utilizing MOFs as sacrificial templates and provides a pathway for exploring high-performance anode materials featuring dual/multi-active centers. Furthermore, the facile strategy for synthesizing MOF-derived polymetallic oxides could be used to facilitate rational design in advanced anode materials for future energy storage, thereby substantially enhancing the lithium storage performance of LIBs.

## Data Availability

The original contributions presented in this study are included in the article and Supplementary Materials. Further inquiries can be directed to the corresponding authors.

## Supplementary Materials

The following supporting information can be downloaded at: https://www.mdpi.com/article/10.3390/molecules31050855/s1, Figure S1: The color comparison of ZIF precursors with different Co/Fe/Ni ratios: (a) **CFN413-ZIF**, (b) **CFN422-ZIF**, and (c) **CFN431-ZIF**; Figure S2: The color comparison of (a) **CFNO422**, **CFNO413** and **CFNO431**; (b) The color of **CFNO422**; Figure S3: The refinement results of XRD for (a) **CFNO422**, (b) **CFNO413** and (c) **CFNO431**; Figure S4: Pore size distribution curves of (a) **CFNO422**, (b) **CFNO413** and (c) **CFNO431**; Figure S5: The SEM images of the precursor (a) **CFN422-ZIF**, (b) **CFNO413**, and (c) **CFNO431**, respectively; Figure S6: The EDS spectrum of **CFNO422**, **CFNO413** and **CFNO431**; Figure S7: The galvanostatic charge/discharge (GCD) curves acquired for the initial three loops at a current density of 0.2 A g^−1^ within a voltage of 0.01–3.0 V of (a) **CFNO422**, (b) **CFNO413**, and (c) **CFNO431**; Figure S8: The relationship between the logarithm of peak current and the scan rate of (a) **CFNO422**, (b) **CFNO413** and (c) **CFNO431**; Figure S9: The separation of pseudocapacitance contribution at 1.0 mV s^−1^ for (a) **CFNO422**, (b) **CFNO413** and (c) **CFNO431**; Figure S10: Profile of a single-step GITT titration of (a) **CFNO413**, (b) **CFNO422**, and (c) **CFNO431**; Table S1: Comparison of Specific surface area of multi-metal oxides anodes in lithium-ion batteries [75,76,77,78]; Table S2: Comparison of lithium storage capabilities of multi-metal oxides anodes [79,80,81,82].
