# Supplementary material for: Trimetallic Zeolitic Imidazolate Framework-Derived CoNiO2/NiCo2O4/NiFe2O4 Hierarchical Architecture: Unveiling Multi-Component Synergism for Ultrahigh-Capacity and Highly Stable Lithium Storage
Source: Molecules. 2026 Mar 4;31(5):855. doi: 10.3390/molecules31050855 (PMC12985757; doi:10.3390/molecules31050855)
Supplement: Supplementary file 1 [file molecules-31-00855-s001.zip › molecules-4119909-supplementary.pdf]

Supporting Information

Trimetallic Zeolitic Imidazolate Framework-Derived $CoNiO_2/NiCo_2O_4/NiFe_2O_4$ Hierarchical Architecture: Unveiling Multi-Component Synergism for Ultrahigh-Capacity and Highly Stable Lithium Storage

Dingyuan Hu [1,†], Ningbo Yu [2,†], Wei Hua [1,*], Xuanyi Gao [2], Yuhong Luo [2,*], Yongbo Wu [3,4], Dong Shu [1,2,*] and Lipeng Zhang [1]

[1] School of Materials and New Energy, South China Normal University, Shanwei 516600, China
[2] School of Chemistry, South China Normal University, Guangzhou 510006, China
[3] Key Laboratory of Atomic and Subatomic Structure and Quantum Control (Ministry of Education), Guangdong Basic Research Center of Excellence for Structure and Fundamental Interactions of Matter, School of Physics, South China Normal University, Guangzhou 510006, China
[4] Guangdong Provincial Key Laboratory of Quantum Engineering and Quantum Materials, Guangdong-Hong Kong Joint Laboratory of Quantum Matter, South China Normal University, Guangzhou 510006, China
* Correspondence: huawei@m.scnu.edu.cn (W. H.); luoyh@scnu.edu.cn (Y. L.); dshu@scnu.edu.cn (D. S.)
† These authors contributed equally to this work

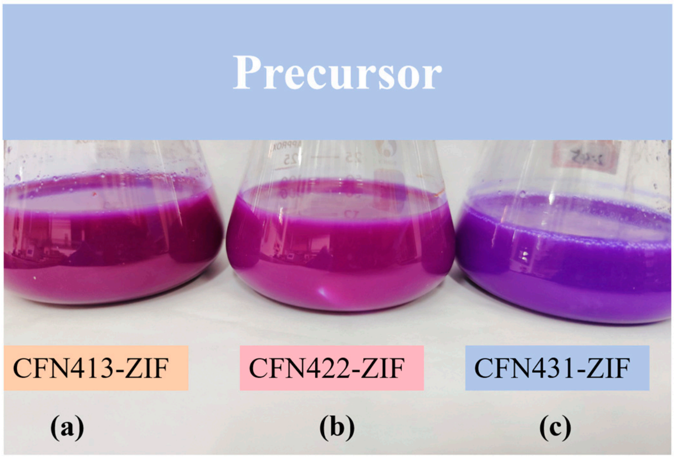

Figure S1. The color comparison of ZIF precursors with different Co/Fe/Ni ratios: (a) **CFN413-ZIF**, (b) **CFN422-ZIF** and (c) **CFN431-ZIF**.

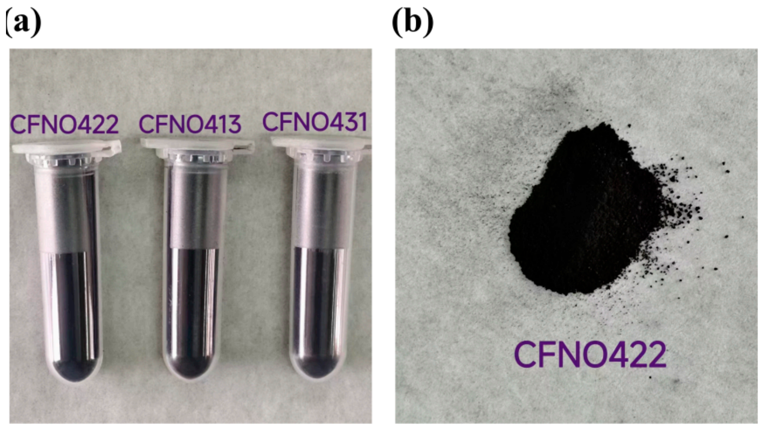

Figure S2. The color comparison of (a) **CFNO422**, **CFNO413** and **CFNO431**; (b) The color of **CFNO422**.

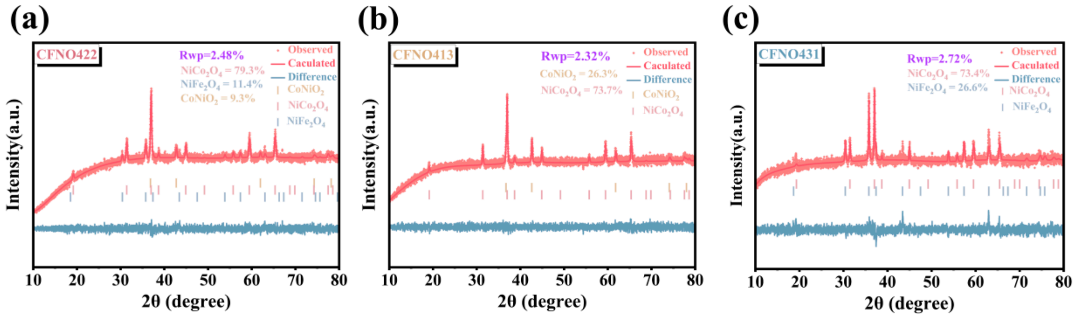

Figure S3. The refinement results of XRD for (a) **CFNO422**, (b) **CFNO413** and (c) **CFNO431**.

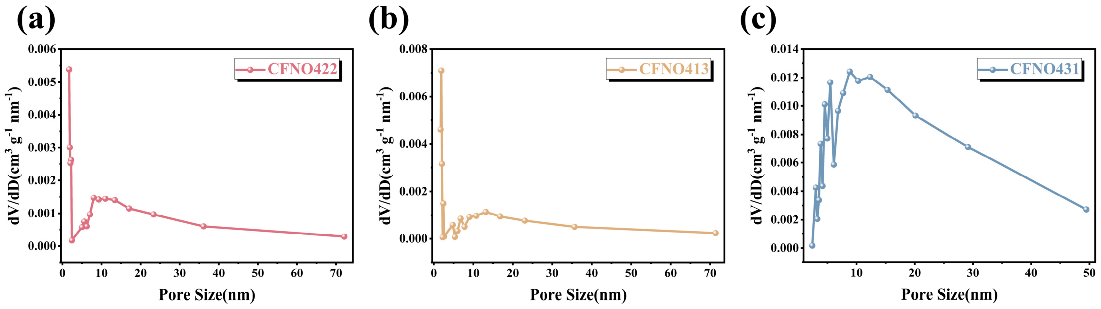

Figure S4. Pore size distribution curves of (a) **CFNO422**, (b) **CFNO413** and (c) **CFNO431**.

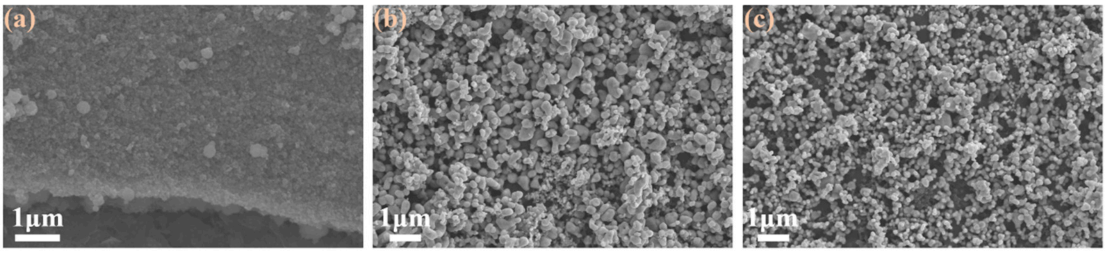

Figure S5. The SEM images of the precursor (a) **CFN422-ZIF**, (b) **CFNO413** and (c) **CFNO431**, respectively.

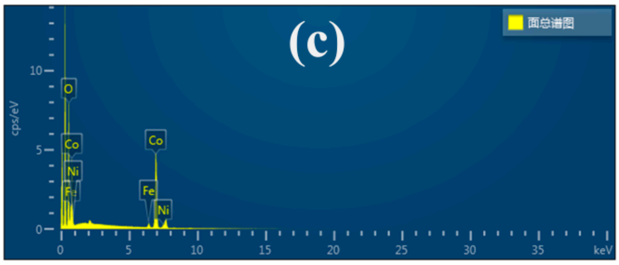

元素	wt%	wt% Sigma	原子百分比
O	38.55	0.24	69.74
Fe	2.46	0.10	1.27
Co	56.71	0.26	27.86
Ni	2.28	0.16	1.13
总量:	100.00		100.00

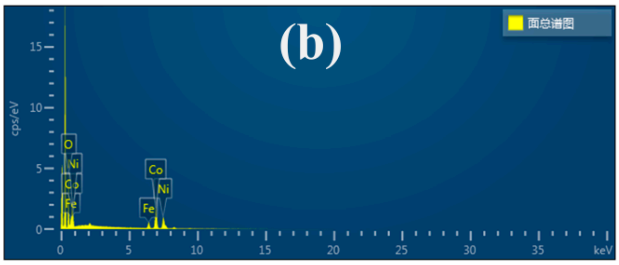

元素	wt%	wt% Sigma	原子百分比
O	38.46	0.36	69.56
Fe	6.87	0.19	3.56
Co	34.40	0.35	16.89
Ni	20.27	0.34	9.99
总量:	100.00		100.00

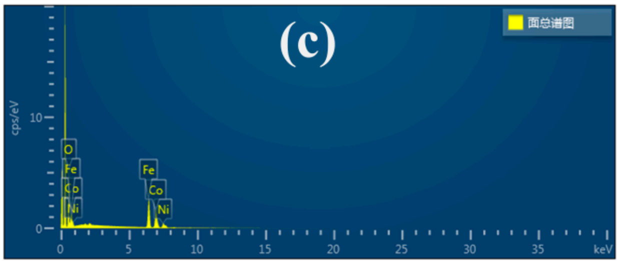

元素	wt%	wt% Sigma	原子百分比
O	36.12	0.16	66.96
Fe	31.81	0.15	16.89
Co	24.90	0.16	12.53
Ni	7.16	0.12	3.62
总量:	100.00		100.00

Figure S6. The EDS spectrum of **CFNO422**, **CFNO413** and **CFNO431**.

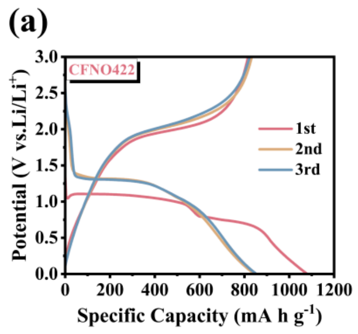

Figure S7. The galvanostatic charge/discharge (GCD) curves acquired for the initial three loops at a current density of 0.2 A g^{-1} within a voltage of 0.01–3.0 V of (a) **CFNO422**, (b) **CFNO413** and (c) **CFNO431**.

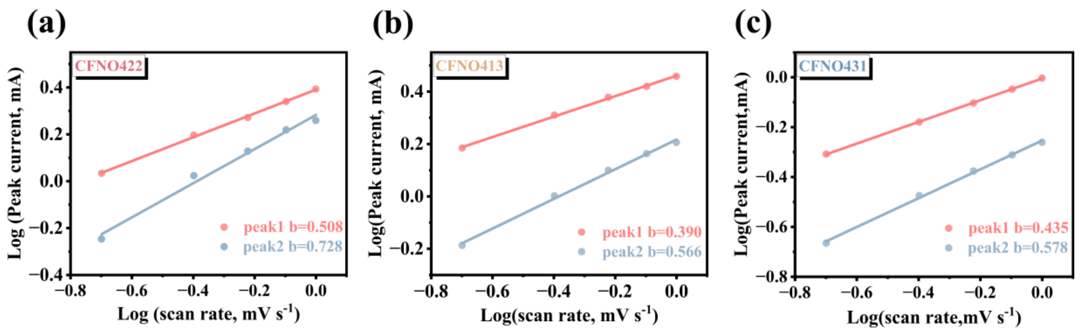

Figure S8. The relationship between the logarithm of peak current and the scan rate of (a) **CFNO422**, (b) **CFNO413** and (c) **CFNO431**.

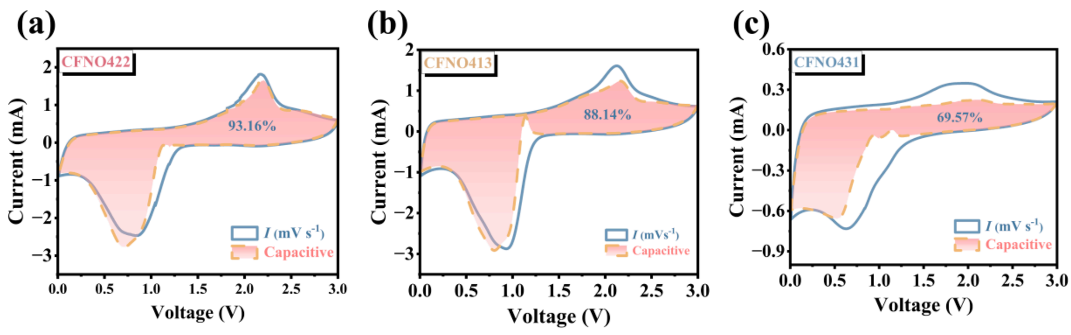

Figure S9. The separation of pseudocapacitance contribution at 1.0 mV s^{-1} for (a) **CFNO422**, (b) **CFNO413** and (c) **CFNO431**.

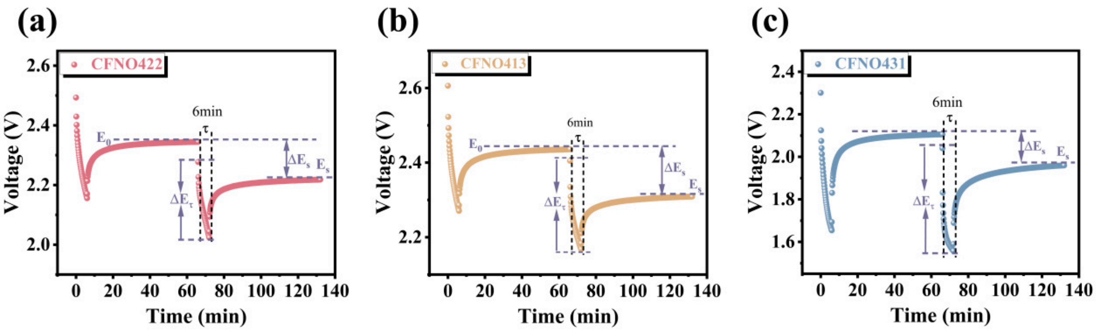

Figure S10. Profile of a single-step GITT titration of (a) **CFNO422**, (b) **CFNO413** and (c) **CFNO431**.

Table S1. Comparison of Specific surface area of multi-metal oxides anodes in lithium-ion batteries.

Samples	Specific surface area (m^2 g^{-1})	Ref.
$ZnMnO_4$	34.62	[S1]
$Mn_{0.5}Zn_{0.5}Co_2O_4$	69.19	[S2]
$NiCo_2O_4$/NC	41.5	[S3]
Mo doped $NiCo_2O_4$	60.32	[S4]
$CoFe_2O_4$	76.4	[S5]
**CFNO422**	102.56	This work

Table S2. Comparison of lithium storage capabilities of multi-metal oxides anodes.

Samples	Current density (A g^{-1})	Cycle number	Capacity (mAh g^{-1})	Ref.
$ZnFe_2O_4$@MnO_2	0.5	100	605.6	[S6]
$Zn_{0.5}Mn_{0.5}Co_2O_4$/RGO	0.1	100	1173.4	[S7]
$NiCo_2V_2O_4$@NC	0.2	100	1189.7	[S8]

$(FeCoNiCrMn)_3O_4$	0.5	300	402	[S9]
$Fe_2O_3/ZnFe_2O_4$	0.1	200	864.8	[S10]
$NiCo_2O_4$/NC	0.1	100	774.8	[S3]
$Ni/CoFe_2O_4$	0.1	50	962	[S12]
$TiO_2@NiCo_2O_4@Co_3O_4$	0.1	200	852	[S13]
**CFNO422**	0.2	120	1301.3	This work

Reference

[S1] Cheng, S.; Ru, Q.; Gao, Y.; Zhen, M; Chen, F.; Wei, L.; Ling, F. Anionic defect-enriched $ZnMn_2O_4$ nanorods with boosting pseudocapacitance for high-efficient and durable Li/Na storage. *Chem. Eng. J.* **2021**, *604*, 126133.

[S2] Ren, Y.; Li, X.; Wang, Y.; Gu, S.; Yang, C.; Gao, T.; Cao, P.; Zhou, G. Preparation of yolk-double shell $Mn_{0.5}Zn_{0.5}Co_2O_4$/C nanomaterials as anodes for high–performance lithium–ion batteries. *Appl. Mater. Today.* **2022**, *27*, 101452.

[S3] Xie, L.; Xu, J.; Liu, M.; Han, Q.; Qiu, X.; Liu, J.; Zhu, L.; Cao, X. Ni-Co MOF-derived rambutan-like $NiCo_2O_4$/NC composite anode materials for high-performance lithium storage. *J. Alloy. Compd.* **2024**, *987*, 174221.

[S4] Ren, Y.; Li, X.; Wang, Y.; Gong, Q.; Gun, S.; Gao, T.; Sun, X.; Zhou, G. Self-template formation of porous yolk-shell structure Mo-doped $NiCo_2O_4$ toward enhanced lithium storage performance as anodematerial. *J. Mater. Sci. Technol.* **2022**, *102*, 186-194.

[S5] Wang, W.; Yao, X.; Ma, Z.; Zhou, J.; Lu, Z.; Sun, J.; Cui, J.; Sha, J. NC coating MOF-derived $CoFe_2O_4$/C spinel for high performancelithium-ion batteries anode. *Chem. Eng. J.* **2025**, *515*, 163912.

[S6] Huang, Y.; Wang, M.; Zhu, Y.; Feng, X; Ding, L.; Guang, Z.; Li, Y.; Zhang, H.; Zhang, N. A novel flower-like metal-based oxides with cross-linked networks for rapid lithium-ion storage. *Int. J. Energy Res.* **2020**, *44*, 4910-4918.

[S7] Yang, C.; Li, X.; Gao, T.; Gu, S.; Wang, X.; Wang, Y.; Wang, Q.; Sun, B.; He, Y.; Zhou, G. Novel quadruple-shelled hollow $Zn_{0.5}Mn_{0.5}Co_2O_4$/RGO heterostructure enable rapid and stable lithium storage performance. *Chem. Eng. J.* **2023**, *474*, 145818.

[S8] Li, X.; Liu, Z.; Zhang, Y.; Li, N.; Zhang, D.; Zhao, S.; Zhao, Y.; $NiCo_2V_2O_8$@NC spheres with mesoporous yolk-bilayer hierarchical structure for enhanced lithium storage. *Langmuir* **2024**, *40*, 15161-15170.

[S9] Wang, D.; Jiang, S.; Duan, C.; Mao, J.; Dong, Y.; Dong, K.; Wang, Z.; Luo, S.; Liu, Y.; Qi, X. Spinel-structured high entropy oxide $(FeCoNiCrMn)_3O_4$ as anode towards superior lithium storage performance. *J. Alloy. Compd.* **2020**, *844*, 156158.

[S10] Fu, Y.; Qiu, W.; Huang, H.; Huang, Q.; Guo, Y.; Mai, W.; Luo, Y.; Xu, Z.; Wu, Y.; Lin, X. Bimetal-

organic framework-templated Zn-Fe-based transition metal oxide composites through heterostructure optimization to boost lithium storage. *J. Colloid Interface Sci.* **2025**, *683*, 507-520.

[S11] Xie, L.; Xu, J.; Liu, M.; Han, Q.; Qiu, X.; Liu, J.; Zhu, L.; Cao, X. Ni-Co MOF-derived rambutan-like $NiCo_2O_4$/NC composite anode materials for high-performance lithium storage. *J. Alloy. Compd.* **2024**, *987*, 174221.

[S12] Xia, S.; Huang, W.; Shen, X.; Liu, J.; Cheng, F.; Guo, H.; Liu, J. Fabrication of porous Ni/$CoFe_2O_4$@C composite for pseudocapacitive lithium storage. *J. Alloy. Compd.* **2021**, *854*, 157177.

[S13] Ye, L.; Yuan, Y.; Zhang, D.; Zhu, M.; Yin, S.; Chen, Y.; Guo, S. Heterogeneous triple-shelled TiO_2@$NiCo_2O_4$@Co_3O_4 nanocages asimproved performance anodes for lithium-ion batteries. *Mater. Lett.* **2018**, *232*, 228-231.
